# Developmental Toxicity of Zinc Oxide Nanoparticles to Zebrafish (*Danio rerio*): A Transcriptomic Analysis

**DOI:** 10.1371/journal.pone.0160763

**Published:** 2016-08-09

**Authors:** Jin Soo Choi, Ryeo-Ok Kim, Seokjoo Yoon, Woo-Keun Kim

**Affiliations:** 1 Future Environmental Research Center, Korea Institute of Toxicology, Jinju, 660–844, Republic of Korea; 2 System Toxicology Research Center, Korea Institute of Toxicology, Daejeon, 305–343, Republic of Korea; Institute of Materials Science, GERMANY

## Abstract

Zinc oxide nanoparticles (ZnO NPs) are being utilized in an increasing number of fields and commercial applications. While their general toxicity and associated oxidative stress have been extensively studied, the toxicological pathways that they induce in developmental stages are still largely unknown. In this study, the developmental toxicity of ZnO NPs to embryonic/larval zebrafish was investigated. The transcriptional expression profiles induced by ZnO NPs were also investigated to ascertain novel genomic responses related to their specific toxicity pathway. Zebrafish embryos were exposed to 0.01, 0.1, 1, and 10 mg/L ZnO NPs for 96 h post-fertilization. The toxicity of ZnO NPs, based on their Zn concentration, was quite similar to that in embryonic/larval zebrafish exposed to corresponding ZnSO_4_ concentrations. Pericardial edema and yolk-sac edema were the principal malformations induced by ZnO NPs. Gene-expression profiling using microarrays demonstrated 689 genes that were differentially regulated (fold change >1.5) following exposure to ZnO NPs (498 upregulated, 191 downregulated). Several genes that were differentially regulated following ZnO NP exposure shared similar biological pathways with those observed with ZnSO_4_ exposure, but six genes (*aicda*, *cyb5d1*, *edar*, *intl2*, *ogfrl2* and *tnfsf13b*) associated with inflammation and the immune system responded specifically to ZnO NPs (either in the opposite direction or were unchanged in ZnSO_4_ exposure). Real-time reverse-transcription quantitative polymerase chain reaction confirmed that the responses of these genes to ZnO NPs were significantly different from their response to ZnSO_4_ exposure. ZnO NPs may affect genes related to inflammation and the immune system, resulting in yolk-sac edema and pericardia edema in embryonic/larval developmental stages. These results will assist in elucidating the mechanisms of toxicity of ZnO NPs during development of zebrafish.

## Introduction

Zinc oxide nanoparticles (NPs) have been used in a wide range of commercial processes and industrial products in recent years (e.g., plastics, ceramics, glass, cement, rubber, paints, pigments, foods, batteries, and fire retardants) [1]. As a result of the widespread use and expanding production of ZnO NPs, the potential for their release into the environment is increasing, potentially leading to toxicity to organisms in ecosystems, and to humans.

The solubility of NPs is crucial to the toxicity of these NPs and to their impacts on ecosystems [2, 3]. It has been suggested that their high stability may allow NPs to permeate, accumulate and persist within organisms [2, 3, 4]. ZnO NPs are easily bioaccumulated by aquatic organisms, wherein they elicit toxic effects [5].

Zebrafish are commonly used as model animals in high-throughput acute toxicity studies, and their value in nanotoxicity assessment has been assessed [6, 7]. Zebrafish are small, transparent, and easy to maintain. They exhibit rapid embryogenesis and continuous reproduction [5]. Importantly, the zebrafish genome has been sequenced and genetic information on this species is rapidly accumulating [8]. Several studies have reported that ZnO NPs are toxic to zebrafish [5, 9, 10]. Zhu et al. demonstrated that ZnO NPs induce a concentration-dependent decrease in hatching rates. Pericardial edema and malformations were observed in ZnO NP-exposed embryos [9]. In addition, previous studies have investigated oxidative stress induced by ZnO NPs in aquatic ecosystems, but their toxicity mechanisms and specific gene biomarkers remain unknown [3, 11, 12].

Although a number of studies have investigated the toxic effects of ZnO NPs on aquatic ecosystems, their effects on aquatic organisms and their toxicity mechanisms are incompletely understood. This study investigates the developmental toxicity of ZnO NPs to embryonic/larval zebrafish. Changes in gene transcription following exposure to ZnO NPs and to ZnSO_4_ are compared to elucidate the toxic mechanism of insoluble zinc, based on the expression levels of specific biomarker genes. The Agilent Zebrafish Oligo Microarray was utilized to investigate the transcriptional responses of zebrafish larvae to ZnO NPs. We analyzed gene expression profiles using the Kyoto encyclopedia of genes and genomes (KEGG) to investigate functional pathways. Microarray data were validated by reverse-transcription quantitative polymerase chain reaction (RT-qPCR). The combination of a well-established model organism with a microarray platform of a large number of genes provides a detailed insight into the molecular mechanisms underlying the adaptive response of fish to ZnO NPs and aids identification of ZnO NP-specific and Zn ion-specific genes and signaling pathways in fish.

## Materials and Methods

### Test chemicals and fish embryos

**Fig 1:**
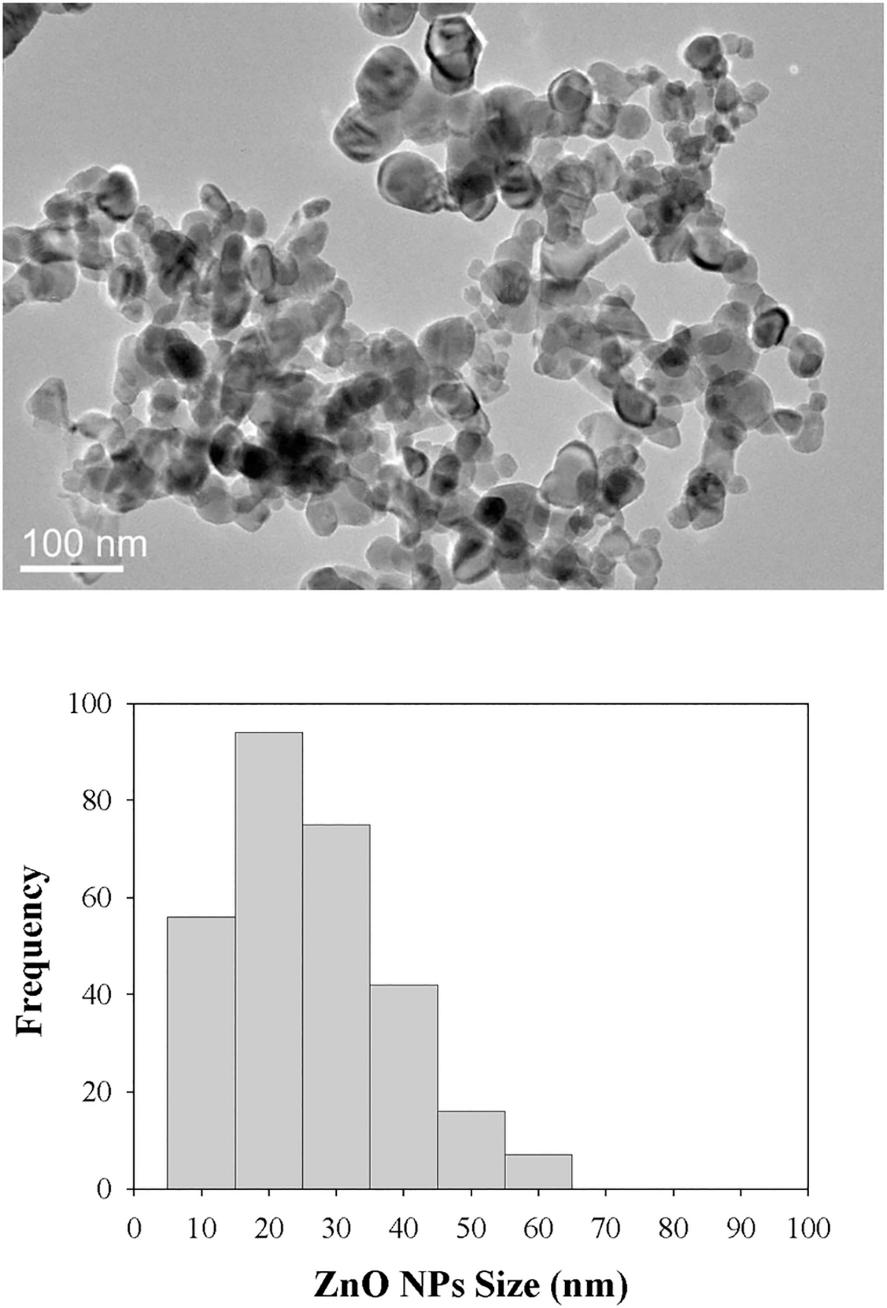
TEM morphology (A) and particle size distribution histogram (B) of zinc oxide nanoparticles.

Zinc sulfate (ZnSO_4_, 99% pure) was obtained from Sigma-Aldrich (St. Louis, MO, USA). ZnO NPs with nominal sizes of 20–30 nm were purchased from SkySpring Nanomaterials (Houston, TX, USA). The physicochemical parameters of ZnO NPs were provided by the company and are shown in Fig 1. The morphology of ZnO NPs was determined by transmission electron microscopy (TEM) at 200 kV (JEM-2010, JEOL, Tokyo, Japan). A solution of 100 mg/L ZnO NPs (mass concentration of the particles) was prepared by dispersing the NPs in DI water for 1 h in a sonicator at 53 W (Sonics, Sonics & Materials Inc., Newtown, CT, USA). Particle size and zeta-potential analysis were performed by dynamic light scattering (Malvern Instruments Ltd., Worcestershire, UK). Zinc concentrations were quantified using an inductively coupled plasma optical emission spectrometer (equipped with S10 Auto sampler, PerkinElmer, Waltham, MA, USA). The concentrations of the test substances are expressed as arithmetic means of their mass concentrations.

Zebrafish (*Danio rerio*) embryos were obtained from the Korea Institute of Toxicology Gyeongnam, Department of Environmental Toxicology and Chemistry. The embryos were incubated at 26°C in zebrafish culture water (carbon-filtered dechlorinated tap water). Embryos at ~2 h post fertilization (hpf) were randomly selected and maintained in 100-mm diameter plastic Petri dishes (100 larvae/dish) containing 10 mL of zebrafish culture water.

### Embryonic toxicity

The fish embryo acute toxicity test (FET) was performed according to OECD test guideline No. 236 [13]. Healthy embryos were placed in 96-well culture plates (1 embryo in 200 μL solution/well). Three replicate 96-well plates were included, each containing 20 wells with each of four concentrations of ZnO NPs test solutions (0.01, 0.1, 1 and 10 mg/L) and 16 wells containing culture medium as the control. The plates were placed in an incubator at 26 ± 1°C with a 14/10 light/dark photoperiod. To determine the relative toxicity of ZnO NPs, ZnSO_4_ exposures were conducted in the same way as ZnO-NP exposures.

The developmental status of the zebrafish embryos and larvae was observed at specified time points (24, 48, 72 and 96 hpf). Hatching and survival rates (percentages) were determined from the total numbers of living embryos remaining. Embryonic malformations were observed using a microscope (Nikon Eclipse Ti, Tokyo, Japan), and the percentage of abnormal embryos was estimated every 24 h.

### Total RNA extractions

Fifty embryos were used to perform the gene-expression experiment in a beaker (250 mL) containing 200 mL of zebrafish culture water. Suspensions of ZnO NPs were introduced to the zebrafish culture water to reach final concentrations of 0 (control) or 2.64 mg/L (LC_25_). In addition, to compare the toxic effects of zinc ions with those of ZnO NPs, reference treatments of 7.75 mg/L ZnSO_4_ (LC_25_) were used. Each treatment was carried out in triplicate. For microarray analysis, the zebrafish embryos were exposed to ZnO NPs for 96 hpf and all zebrafish larvae were anesthetized in 100 mg/L of tricaine methanesulfonate (MS-222; Sigma-Aldrich, St. Louis, MO, USA) following guidelines of the Institutional Animal Care and Use Committee (IACUC) of the Korea Institute of Toxicology.

The total RNA from ~50 zebrafish larvae cultured together in the same dish was extracted using an RNeasy mini kit (Qiagen, Hilden, Germany). RNA integrity was confirmed by determining the RNA integrity number with an Agilent Bioanalyzer 2100 (Agilent RNA 6000 Nano Kit Guide, Agilent Technologies, Santa Clara, CA, USA).

### RNA labeling and microarray

A one-color microarray analysis of the gene expression levels in zebrafish larvae was performed by NEX BiO (Daejeon, the Republic of Korea) using the Agilent Zebrafish Oligo Microarray (4 × 44K) containing 43,803 *Dario rerio* probes. Three replicate RNA samples from the LC_25_ ZnO NP (2.64 mg/L) and LC_25_ ZnSO_4_ (7.75 mg/L) treatments were independently collected, and control treatments were conducted. All of the steps, from the RNA labeling to the scanning steps, were performed according to the protocol for One-Color Microarray-Based Gene Expression Analysis (Low Input Quick Amp Labeling; Agilent Technologies, Wilmington, DE, USA) with slight modifications. The high-quality RNA samples were purified using the RNeasy mini kit (Qiagen).

The total RNA was amplified with the Low RNA Input Linear Amplification kit (Agilent Technologies, Wilmington, DE, USA) and labeled with 1.65 μg of Cy3 according to the manufacturer’s instructions. The labeled cRNA was purified with the RNeasy mini kit (Qiagen). Each slide was hybridized with Cy3-labeled cRNA using the Gene Expression Hybridization Kit (Agilent Technologies, Wilmington, DE, USA). After hybridization for 17 h, the slides were washed with the stabilization and drying solution in the Gene Expression Wash Buffer Kit (Agilent Technologies, Wilmington, DE, USA).

The microarray slides were scanned with an Agilent Scanner B, and the signal intensities were analyzed using the Feature Extraction software 10.7 with the default settings. The raw data were normalized using the percentile shift method.

### Analysis of microarray data

The expression levels represent the ratios obtained for zebrafish treated with 20–30-nm ZnO NPs and ZnSO_4_ relative to non-treated zebrafish. The expressed genes common to the zebrafish treated with ZnO NPs and ZnSO_4_ were sorted by Venn diagram analysis. Additionally, the treatment-specific response genes were sorted by heatmap analysis (linkage methods: average linkage; similarity/distance measure: Pearson correlation coefficient). Subsequently, the expressed genes that were differentially expressed between the zebrafish treated with ZnO NPs and those treated with ZnSO_4_ were filtered as follows: fold ratio ≥1.5 and ≤−1.5. The functional analysis of the differentially expressed genes (DEGs) from KEGG databases was enriched using Gene Spring GX 11.5.1 (Agilent Technologies, San Clara, CA, USA). The data produced in this study are available from the Gene Expression Omnibus database (GSE77148).

### qRT-PCR

To validate the microarray data, the total RNA was reverse-transcribed using the Superscript® III First-Strand Synthesis System for RT-PCR (Invitrogen, Carlsbad, CA, USA), and the cDNA was amplified with seven primers designed using Primer 3 software (version 4.0). Six genes (*aicda*, *cyb5d1*, *edar*, *intl2*, *ogfr12* and *tnfsf13b*) and *beta actin* (endogenous control gene) were selected from the available sequences at GenBank. The gene accession numbers and primer sequences used for the quantitative real-time PCR are shown in Table C in S1 File. The SYBR Green-based quantitative real-time PCR was performed on an Agilent Mx3005P qPCR system (Agilent Technologies, Santa Clara, CA, USA). The reactions were performed using the Brilliant III Ultra Fast SYBR® Green QPCR Master mix (Agilent Technologies, Foster City, CA, USA) for one cycle of 95°C for 3 min followed by 40 cycles of 95°C for 10 s and 60°C for 20 s.

The relative quantification of gene expression was performed using the 2^∆∆Ct^ method [14]. The efficiencies of the PCR reactions for the genes were 90–110% under the optimized qPCR conditions, and the specificity of the primers was determined through melting-curve analysis [15]. Each amplification reaction was performed in triplicate.

### Statistical analysis

Differences among the treatments were analyzed by one-way analysis of variance (ANOVA) followed by *post hoc* comparisons using least significant difference (LSD) or Tukey’s HSD tests (SPSS v20, IBM, Armonk, NY, USA). Differences between the treatment and control groups were considered significant where *p* <0.05. All data are presented as means ± SEM. The median lethal concentration (LC_25_) and 95% confidence intervals were determined in terms of mass concentrations (ZnO or ZnSO_4_) and measured atomic concentrations (Zn) using a linear regression method (CETIS v1.8.7, Tidepool, Mckinleyville, CA, USA).

## Results and Discussion

### ZnO-NP exposure conditions

The diameters of the ZnO NPs were determined to be approximately 20–30 nm from TEM images (Fig 1). At the end of the tests, the diameters of ZnO NPs at 10 mg/L and 100 mg/L had increased to 242.63 ± 2.70 nm and 288.63 ± 1.58 nm, respectively (Table B in S1 File). As expected, the ZnO NPs agglomerated to form larger particles when dispersed in deionized water. In exposure experiments using nanomaterials, dispersion is an important factor in relation to their toxic effects, which may be affected by size, shape, surface charge, and physicochemical properties [16, 17]. Although ZnO NPs are well dispersed in aqueous solutions compared with other metal NPs, nevertheless, agglomeration can occur and the size increase could affect their toxicity. To optimize particle dispersion and minimize agglomeration, we sonicated the suspensions and analyzed them for changes in agglomeration and zeta potential of the material over time [16]. The concentrations of Zn were measured at the beginning and end of the tests (Table A in S1 File). The final concentrations of ZnO NPs and ZnSO_4_ were consistent with their initial concentrations, indicating that they were essentially constant throughout the exposure period.

### Comparative toxicity of ZnO NPs and ZnSO_4_ to developmental stages of *D*. *rerio*

**Fig 2:**
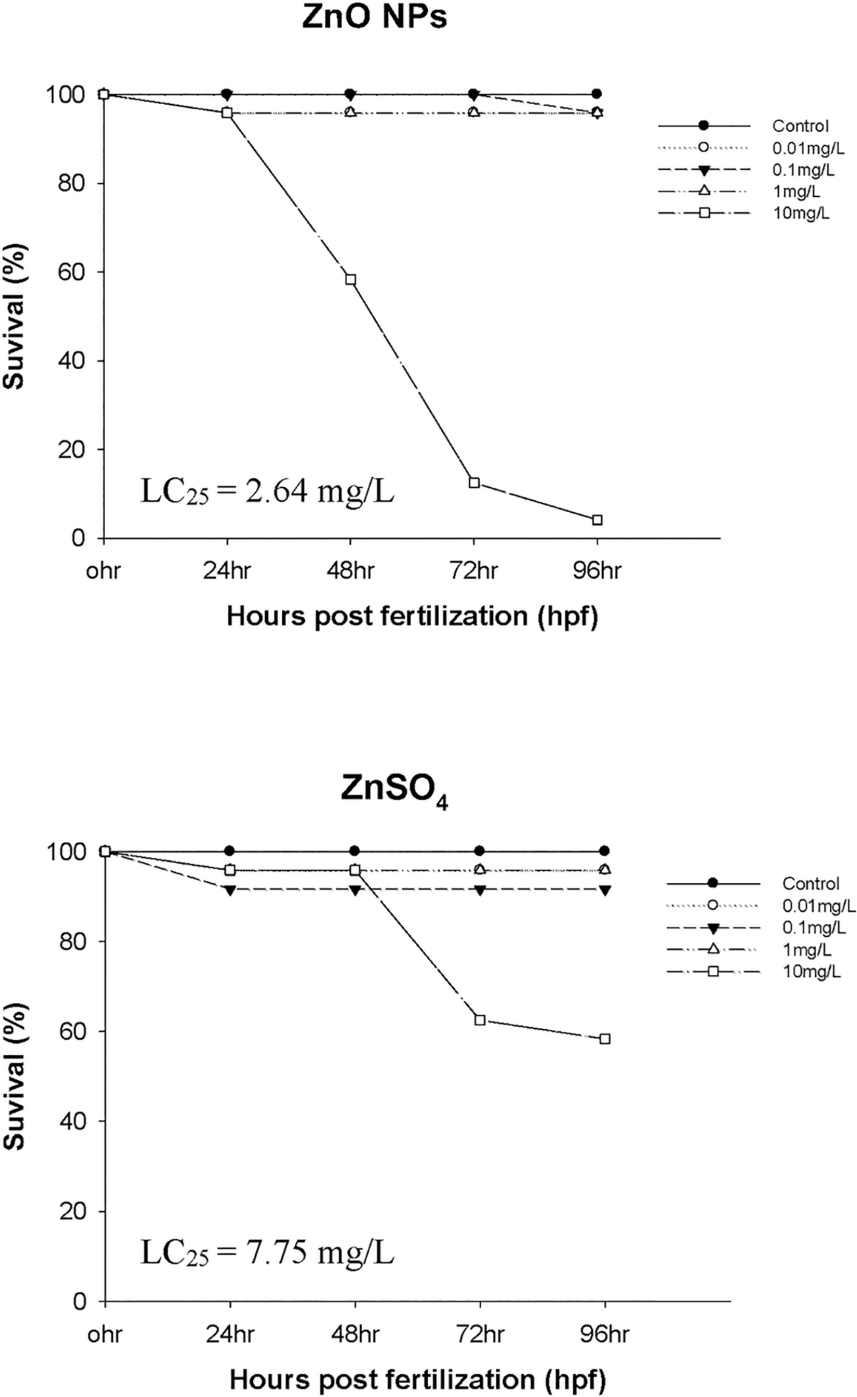
Survival rates of zebrafish embryos exposed to different concentrations of ZnO NPs and ZnSO_4_ for 96 hpf.

**Fig 3:**
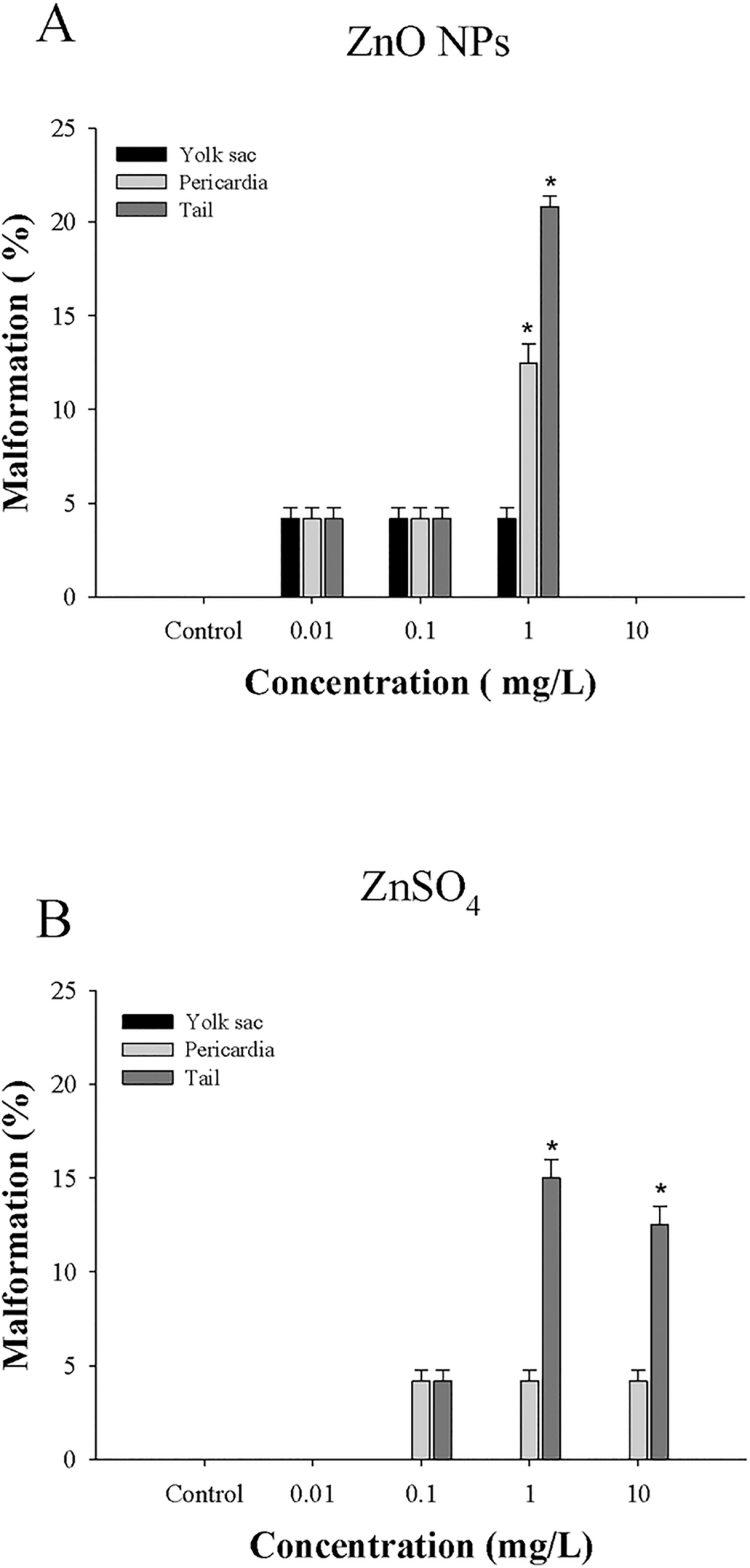
**Malformations of zebrafish embryos exposed to ZnO NPs (A) and ZnSO**_**4**_ **(B).** Pericardial and tail malformations were most frequent in both treatments. Data are expressed as means ± SEM from three independent experiments, n = 8 (*represents a significant difference from controls (one-way ANOVA followed by Tukey’s *post hoc* test, *p*<0.05).

**Fig 4:**
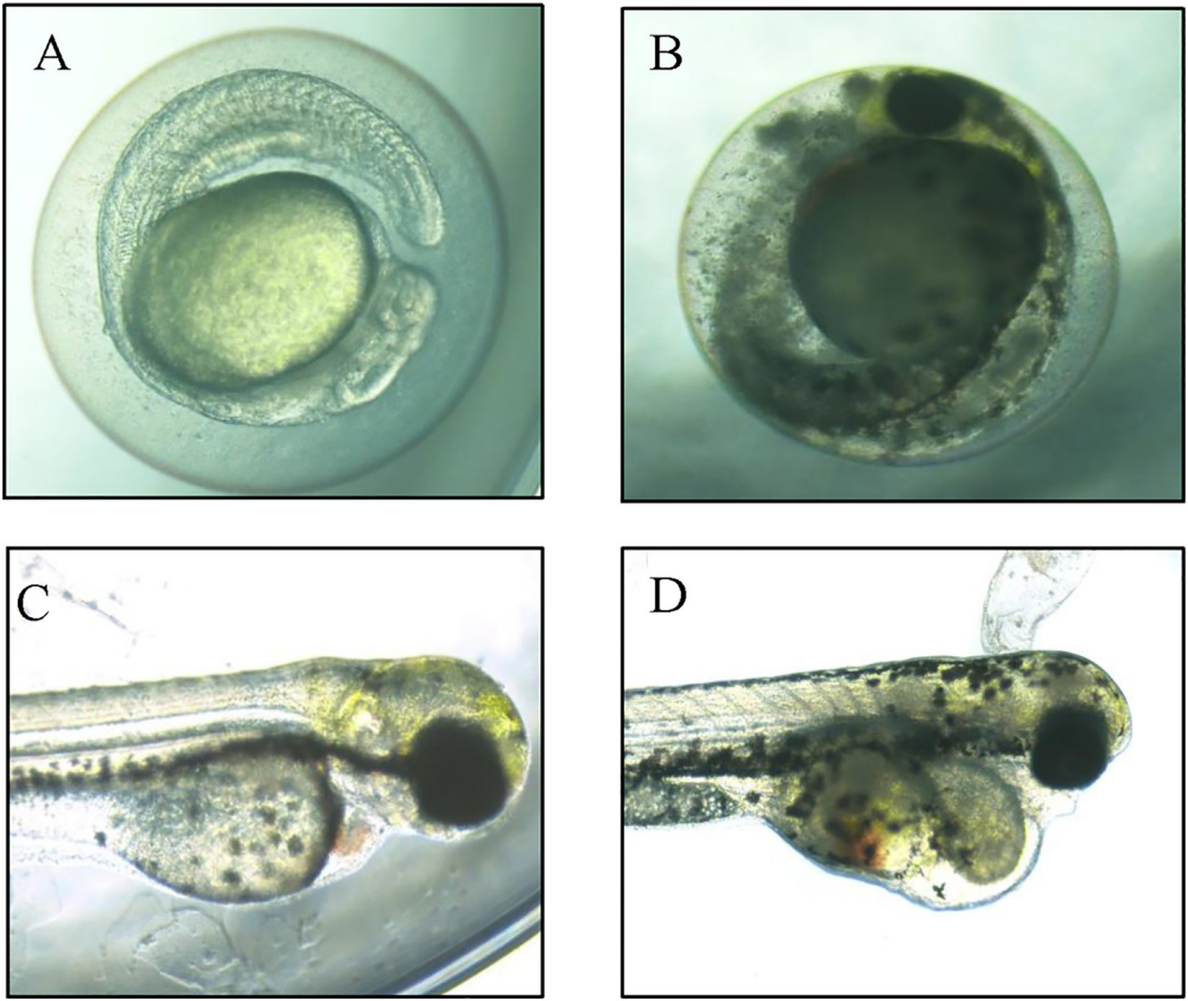
Representative optical images of deformed zebrafish caused by exposure to ZnO NPs. The control group shows the normal appearance at 24 hpf (A) and at 96 hpf (C). Yolk-sac edema (B) and pericardial edema (D) are shown after exposure to 1 mg/L ZnO NPs for 72 and 96 hpf, respectively.

To compare the toxic effects of ZnO NPs and ZnSO_4_ on the development of embryonic-larval zebrafish, sublethal toxicity endpoints were measured, i.e., survival rates (Fig 2) and types of embryonic malformation (Figs 3 and 4). LC_25_ values based on nominal mass concentrations were 2.64 mg/L for ZnO NPs (measured 1.78 mg Zn/L) and 7.75 mg/L for ZnSO_4_ (measured 1.71 mg Zn/L), indicating that, in terms of their mass concentrations, there was a relatively high incidence of embryonic mortality after ZnO NPs exposure (Table 1). This result is consistent with a previous report that *D*. *rerio* was more sensitive to ZnO NPs than ZnSO_4_ based on their mass concentrations [18].

**Table 1:** Comparative toxicity of ZnO NPs and ZnSO_4_ in *Danio rerio*.

Treatment	Concentration of particles/molecules (mg/L)	Measured zinc concentration (mg/L)
	LC_25_ (95% confidence intervals)	LC_25_ (95% confidence intervals)
ZnO NPs	2.64 (0.41–3.90)	1.78 (0.42–32.70)
ZnSO_4_	7.75 (5.13–9.93)	1.71 (0.18–2.91)

Developmental defects including tail malformation, pericardial edema, and yolk-sac edema were observed under exposure to ZnO NPs and ZnSO_4_ (Figs 3 and 4). Interestingly, ZnO NPs simultaneously induced three different types of developmental malformation, in contrast to the effects of other nanomaterials [19]. Specifically, zebrafish embryos exhibited yolk-sac edema (lesions of the yolk sac) at all concentrations ZnO investigated, but no such changes were observed after ZnSO_4_ exposure. The similar toxicities of ZnO NPs and ZnSO_4_ based on their Zn concentrations suggest that a synergistic effect between the NPs and the Zn ions derived from ZnO NPs may lead to their development toxicity in zebrafish [10].

The toxicity of NPs is usually attributed to their small size and high specific surface area [18, 20], and NPs are expected to be more toxic than their bulk counterparts owing to their accumulation within cells and organisms [21, 22, 23]. In contrast, other studies comparing the toxicity of ZnO NPs and Zn^2+^ found the ZnO NPs to be less toxic [24, 25]. Aggregations of NPs with size distributions similar to those of their bulk particles in suspension indicate that the toxicity mechanisms of NPs are complex [18, 26, 27]. Zhou *et al*. reported that ZnO NPs induce oxidative damage, and that intracellular zinc is important in the toxicity of ZnO NPs but it is difficult to separate particle-induced toxicity and the effect of dissolved Zn (2^+^) [28].

### Differentially expressed genes in ZnO NP-exposed larval zebrafish

**Fig 5:**
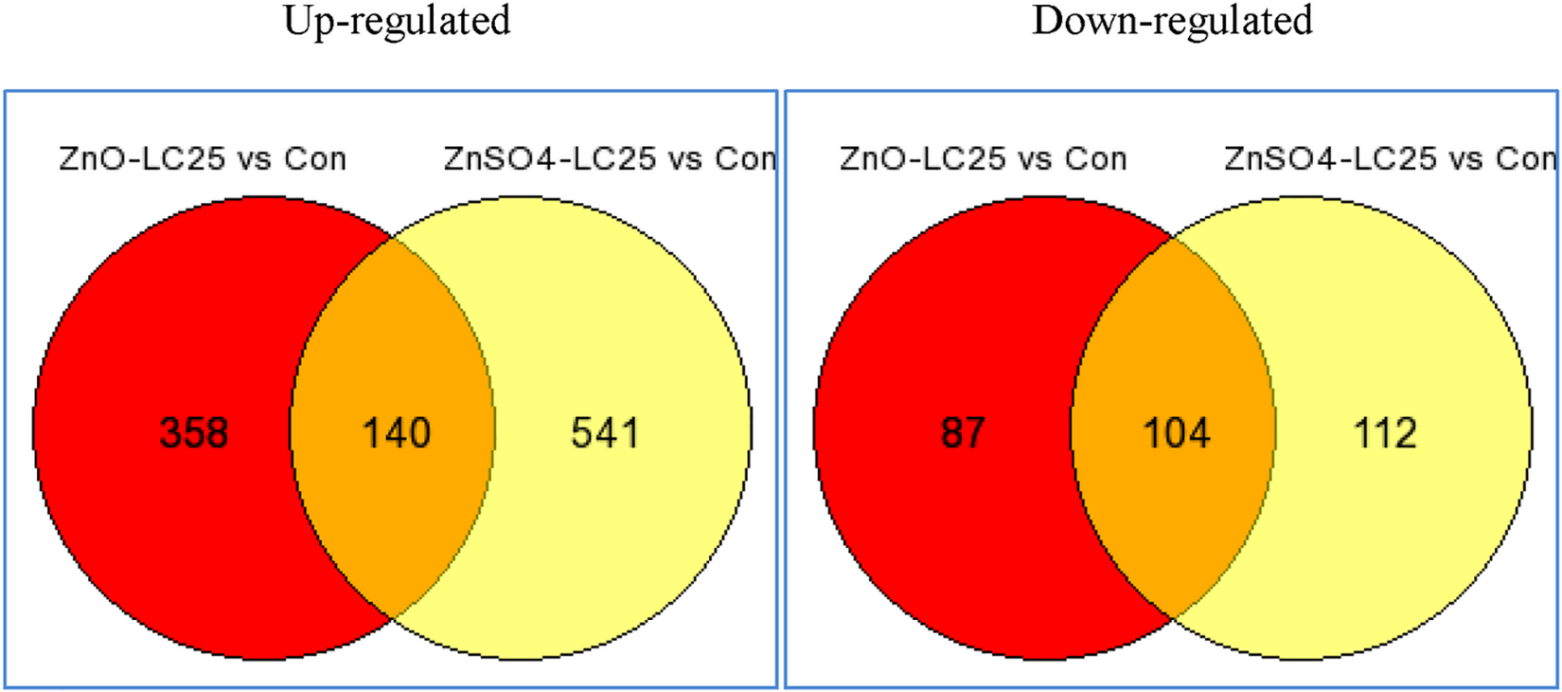
Venn diagram showing numbers of up- and down-regulated genes following exposure to ZnO NPs and ZnSO_4_.

Gene expressions in larval zebrafish exposed to LC_25_ concentrations of ZnO NPs and to ZnSO_4_ were compared with controls. Statistical analysis and subsequent filtering out of genes with low or no signal and a fold change <1.5 revealed 358 up-regulated and 87 down-regulated transcripts associated with ZnO NPs, and 541 up-regulated and 112 down-regulated transcripts with ZnSO_4_ (Fig 5). The relative expression of the genes in zebrafish larvae showed more DEGs in fish exposed to ZnSO_4_ than in those exposed to ZnO NPs (Fig 5), indicating that their respective degrees of transcriptional alteration were not consistent with the levels of their morphological and physiological changes. However, DEGs between ZnO NPs and ZnSO_4_ accounted for 10% of up-regulated and 34% of down-regulated genes of total DEGs, suggesting that the molecular mechanisms responsible for the toxicities of the two forms of Zn differed.

Among the DEGs for ZnO-NP exposure, more were stimulated than suppressed, indicating that the ZnOs represented a severe stress condition for larval zebrafish. Zhao *et al*. reported that their small size helps ZnO NPs to be easily be absorbed into biological systems through cellular uptake and interactions with internal or membrane molecules [29]. The effect of ZnO NPs was reported to result solely from Zn^2+^ ions [30], but other reports suggested that NPs and Zn^2+^ induced effects additional to those of dissolved Zn (II) alone [1, 5, 10, 24, 29]. However, changes in expression after exposure to ZnO NPs and the specific pathways regulated by NPs have not previously been reported [29]. Therefore, abnormalities in embryonic development coupled with whole gene transcriptional profiling could help to elucidate the diverse cellular and molecular events associated with the toxicity of ZnO NPs.

Most of the gene expression changes were associated with both ZnO NPs and ZnSO_4_ treatments, but *ogfrl2* (opioid growth factor receptor-like 2 [*ogfrl2*]) and *cyb5d1* (cytochrome b5 domain containing 1) genes were induced by ZnO NPs LC_25_ to 6.81-fold and 4.12-fold higher levels, respectively (Table A in S2 File). The *ogfrl* gene has not previously been implicated in a response to toxicity in zebrafish. It is an integral membrane protein associated with the outer nuclear envelope, which, after binding to the native opioid peptide [Met5]-enkephalin (termed OGF), inhibits DNA synthesis by inducing the expression of cyclin-dependent kinase inhibitors p16 (INK4a) and p21 (WAF1/CIP1) [31]. Their negative regulation in cell growth has been well demonstrated in cancer cell lines [32, 33]. Also, in mouse macrophages, *ogfr* regulates the expression of IFNγ, suggesting that they might be involved in the proliferation, differentiation and/or effector functions of macrophages [34]. Moreover, it was reported that the *ogfr* gene is up-regulated in the chronic alcohol-exposed zebrafish, associated with behavioral adaptation and neurochemical changes [35]. Therefore, overexpression of ogfrl2 on exposure to ZnO NPs could act as a potential regulator generating morphological defects in zebrafish embryo-larval development. Additionally, the functionally unknown *cyb5d1* gene in zebrafish was significantly up-regulated on ZnO-NP exposure. Recently, it has been identified in several reports as one that is regulated in breast cancer [36, 37]. Thus, further studies will be needed to understand the role of cyb5d1 in embryonic development of zebrafish. Taken together *ogfrl2* and cyb5d1, genes involved in transcriptional regulation (cyclin K [*ccnk*], tudor domain containing 1 [*Tdrd1*]), detoxification (cytochrome b5 domain containing 1 [*cyb5d1*]), and developmental regulation in embryos (mohawk homeobox b [*mkxb*]) were up-regulated, which is suggestive of efforts to initiate developmental processes under stress. These results indicate that ZnO NPs might be involved in ontogenesis and the immune system of zebrafish.

Conversely, *intl2* (Intelectin 2) was significantly reduced by ZnO NPs (−4.32-fold change), but there are no other reports of its expression related to toxicity in zebrafish. The *intl2* gene is a member of the intelectin family, newly discovered genes involved in development and innate immunity [38]. Takano *et al*. reported that *intl1* was expressed in leukocyte cell lines, but no expression of *intl2* was detected in any of the leukocyte cell lines. This may be a consequence of the initial immune response and/or a downstream immune response, rather than being a component of the primary immune responses [39]. In addition, a number of unknown genes *sc*:*d0261*, *stc1l*, *zgc*:*172323*, *LOC100332*, and *arg1* were significantly reduced more than 5-fold in the ZnO NPs. The complete list of genes up-regulated and down-regulated by ZnO-NP and ZnSO_4_ treatments is shown in Table A in S2 File.

### Functional analysis of differentially expressed genes

KEGG pathway analysis revealed ZnO NP-specific toxicological pathways concerned with cytokine-cytokine receptor interactions and the intestinal immune network for IgA production (Table 2). In the cytokine-cytokine receptor interaction pathway, specific genes in related fish models include *edar* (ectodysplasin A receptor) and *tnfsf13b* (tumor necrosis factor (ligand) superfamily, member 13b). The *edar* gene encodes a transmembrane protein with similarity to tumor necrosis factor receptor (TNFR).

**Table 2:** Heatmaps of pathway (KEGG) enrichment analysis for ZnO NP- and ZnSO_4_-regulated genes.

	ZnO-LC_25_	ZnSO_4_-LC_25_
**Up-regulation**		
Cytokine-cytokine receptor interaction	0.029	
Neuroactive ligand-receptor interaction		0.012
Cell adhesion molecules (CAMs)		0.004
Intestinal immune network for IgA production	0.030	
**Down-regulation**		
Circadian rhythm	0.002	0.003

Up- and down-regulated genes with fold changes ≥1.5 or ≤−1.5, respectively (*p* < 0.05). The values represent significance as *p*-values.

EDA signaling involving the *edar* gene is associated with variations in morphology that occurred during the evolution of teleost fishes [40]. In particular, the pathway is necessary for the development and patterning of the dermal bones of the skull, scales, fin rays and teeth of the adult zebrafish [41]. In the light of these functions of the *edar* gene, we suggest that up-regulation of this gene in response to ZnO NPs should be regarded as a component of the regulators responsible for morphological changes in early development and that eventually affect adult body form. The *tnf13b* gene (also known as BAFF) is a member of the TNFSF that plays an important role in B-cell survival, proliferation and differentiation, and with T-cell activation. The association with increased BAFF expression after bacterial and viral infections suggests that it plays a potentially important role in the immune system of fish [42]. *tnfsf13b* expression is significantly increased in lung adenocarcinomas and malignant breast tissue [43, 44].

Under the pathway of the intestinal immune network for IgA production, specific gene-related fish models include the *aicda* (activation-induced cytidine deaminase) gene. The AICDA enzyme is a B lymphocyte-specific member of the apolipoprotein B mRNA-editing catalytic component (APOBEC) family of Zn-dependent cytidine deaminases. In zebrafish embryogenesis, *aicda* knockdowns revealed an increase of abnormalities in neuronal development, and hypermethylation at loci encoding for transcription factors involved in neurogenesis [45]. The cytokine-cytokine receptor interaction pathway is proportional to the number of genes shared by the pathway of the intestinal immune network for IgA production and the hemopoietic cell-lineage pathway and is connected to a network of biological links within the immune pathways [46].

**Fig 6:**
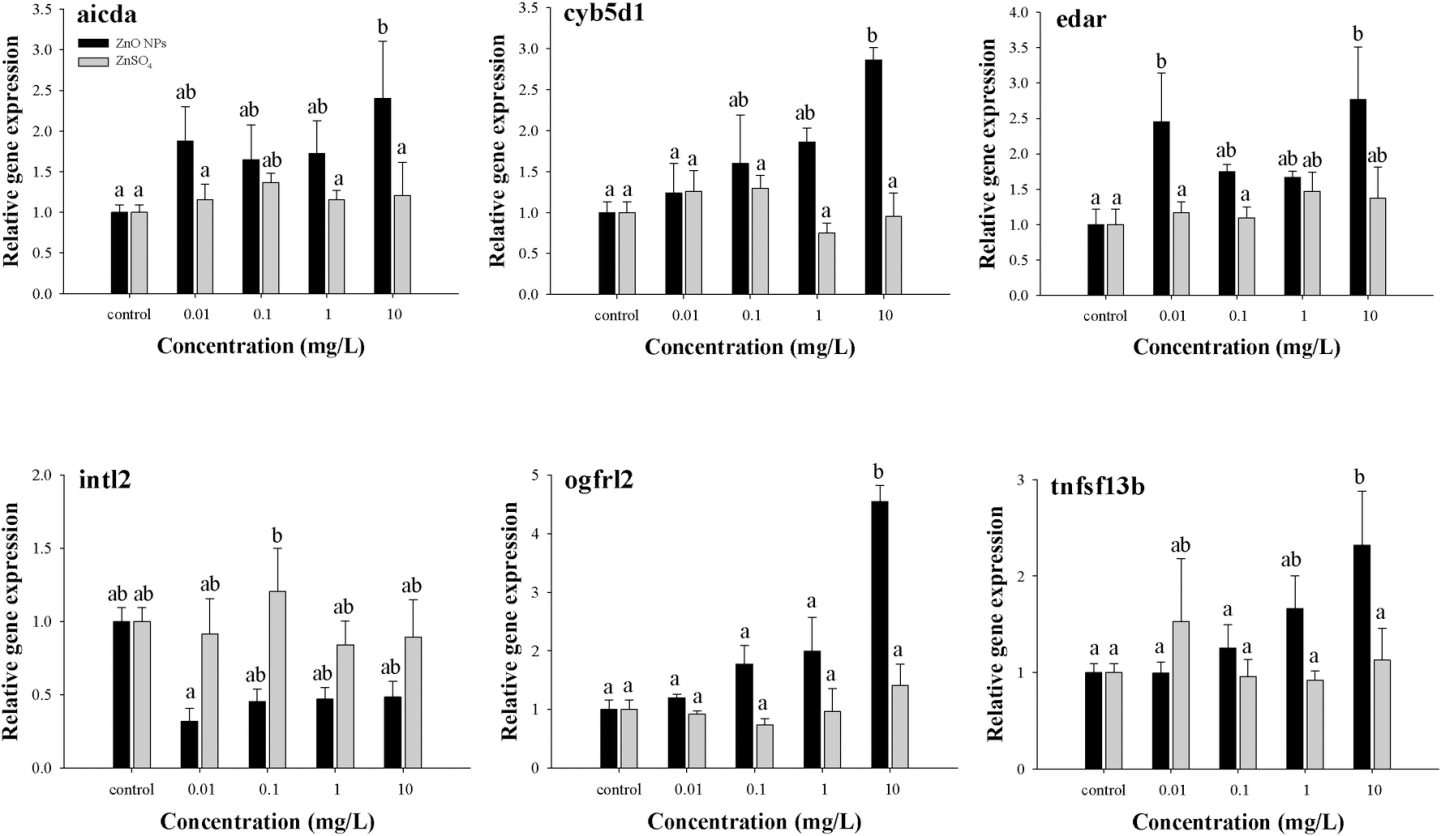
Relative expression of six genes in zebrafish embryos exposed to ZnO NPs and ZnSO_4_ for 96 hpf. Values are presented as means ± SEM (*n* = 3). Significant differences among treatment groups are denoted by different letter labels (one-way ANOVA, followed by a *post hoc* test: Tukey’s (*cyb5d1*, *intl2* and *ogfrl2*) and LSD (*aicda*, *edar*, and *tnfsf13b*), *p* < 0.05).

To confirm the molecular modes of actions caused by exposure to ZnO NPs, three genes that were influenced by the pathways of the intestinal immune network for IgA production (*aicda* and *tnfsf13b*), and cytokine-cytokine receptor interaction (*edar* and *tnfsf13b*), were selected for qPCR analysis (Fig 6). Additionally, the ZnO NP-specific changes in the expression profiles of the *cyb5d1*, *intl2* and *ogfrl2* genes were randomly selected from the up- and down-regulated transcriptional change data (fold change >1.5, *p* < 0.05). The gene expression levels estimated by qPCR showed good agreement with microarray data. The transcriptional levels of the pathway-related genes were increased after ZnO-NP exposure compared with exposure to ZnSO_4_, except in the case of the *intl2* gene.

This study illustrates the value of high-throughput transcriptional data and has identified potential biomarkers that were differentially regulated after exposure to ZnO NPs, irrespective of whether they were differentially expressed after ZnSO_4_ treatment. Our aim was to examine gene expression, molecular mechanisms, related morphological changes and mortality caused by ZnO NP-toxicity in zebrafish. Although many genetic changes were identified, it was difficult to precisely determine their roles. Following exposure to ZnO NPs, we show a number of genetic changes (including putative biomarkers) associated with the cytokine receptor and immune networks that probably contribute to the toxic effects, including morphological changes.

## Conclusions

This study specifically provides insights into the toxicology of ZnO NPs for developmental processes and demonstrates transcriptional responses through microarray analysis. The toxicity of ZnO NPs, based on Zn levels, was quite similar to that in embryonic/larval zebrafish exposed to corresponding ZnSO_4_ concentrations. However, malformations, which included pericardial edema, tail edema, and yolk-sac edema in ZnO NP-exposed developmental stages of zebrafish, differed from those of the ZnSO_4_-exposure group. Specifically, ZnO NPs induced overexpression of *ogfrl2* and *cyb5d1* genes, which have not previously been associated with NP toxicity, and significantly changed expression of genes related to cytokine receptor and immune networks. Therefore, ZnO NPs could cause adverse physiological outcomes resulting from impaired signal transduction. Further studies are still needed to better understand the genes, especially the time-course of their expression following ZnO-NP exposure.

## Supporting Information

S1 File **Table A** Concentrations of ZnO-NPs, ZnSO_4_ and atomic Zn concentrations, (means ± SD) in the ZnO-NP and ZnSO_4_ treatments, initially and after 96 h **Table B.** Size and Z-potential of ZnO nanoparticles (means ± SD) in DI water **Table C.** Primer used in qRT-PCR validation (MS word file).
(DOCX)

S2 File **Table A.** Fold changes of transcripts (up- and down-regulated genes).
(XLSX)
