# Supplementary material for: Developmental Toxicity of Zinc Oxide Nanoparticles to Zebrafish (Danio rerio): A Transcriptomic Analysis
Source: PLoS One. 2016 Aug 9;11(8):e0160763. doi: 10.1371/journal.pone.0160763 (PMC4978389; doi:10.1371/journal.pone.0160763)
Supplement: S1 File — Table A Concentrations of ZnO-NPs, ZnSO4 and atomic Zn concentrations, (means ± SD) in the ZnO-NP and ZnSO4 treatments, initially and after 96 h Table B. Size and Z-potential of ZnO nanoparticles (means ± SD) in DI water Table C. Primer used in qRT-PCR validation (MS word file). (DOCX) [file pone.0160763.s001.docx]

Table A. Concentrations of ZnO-NPs, ZnSO_4_ and atomic Zn concentrations, (means ± SD) in the ZnO-NP and ZnSO_4_ treatments, initially and after 96 h

|  | | Measured (mg/L) | | Dissolved Zn (µg/L) | |
| --- | --- | --- | --- | --- | --- |
| Nominal concentration (mg/L) | | 0 h | 96 h | 0 h | 96 h |
| ZnO NPs | 0.01 | 0.029 ± 0.000 | 0.078 ± 0.000 | 0.000 ± 0.000 | 0.014 ± 0.000 |
|  | 0.1 | 0.158 ± 0.000 | 0.153 ± 0.000 | 0.009 ± 0.001 | 0.044 ± 0.003 |
|  | 1 | 1.062 ± 0.017 | 1.019 ± 0.004 | 0.818 ± 0.005 | 0.774 ± 0.029 |
|  | 10 | 7.431 ± 0.052 | 7.040 ± 0.048 | 5.797 ± 0.642 | 5.694 ± 0.055 |
| ZnSO_4_ | 0.01 | 0.017 ± 0.000 | 0.033 ± 0.001 | 0.001 ± 0.000 | 0.088 ± 0.003 |
|  | 0.1 | 0.081 ± 0.000 | 0.106 ± 0.000 | 0.005 ± 0.002 | 0.017 ± 0.000 |
|  | 1 | 0.507 ± 0.013 | 0.497 ± 0.001 | 0.328 ± 0.029 | 0.310 ± 0.033 |
|  | 10 | 4.657 ± 0.028 | 4.996 ± 0.065 | 6.263 ± 0.171 | - 1. ± 0.076 |

Table B. Size and Z-potential of ZnO nanoparticles (means ± SD) in DI water

|  | | Size (nm) | | Z-potential (mV) | |
| --- | --- | --- | --- | --- | --- |
| Nominal (mg/L) | | 0 h | 96 h | 0 h | 96 h |
| ZnO NPs | 1 | n.d | n.d | n.d | n.d |
|  | 10 | 202.56 ± 23.46 | 242.63 ± 2.70 | 18.90 ± 0.70 | 19.00 ± 0.50 |
|  | 100 | 203.23 ± 2.54 | 288.63 ± 1.58 | 25.13 ± 0.76 | 21.50 ± 0.40 |

*n.d: not detected.

Table C. Primers used in qRT-PCR validation

| Probe Name | Gene Symbol | Forward sequence | Reverse sequence | Ref Seq Accession | product size |
| --- | --- | --- | --- | --- | --- |
|  |  |  |  |  |  |
| A_15_P6311 | *aicda* | ctgtcacagacgcccaatct | aaaactccgctccctccttg | NM_001008403 | 189 |
| A_15_P6583 | *cyb5d1* | aggtgtcgcttcacaacaca | ctttgggtcgaaccagtgac | BC155725 | 158 |
| A_15_P2022 | *edar* | cgcgtcatcagaaaacgagc | cttctgctgtgaatcccggt | NM_001115064 | 195 |
| A_15_P1444 | *intl2* | cctgctgctccaatcgtgta | tagggaccccctcaggaaag | NM_001159541 | 220 |
| A_15_P1359 | *ogfrl2* | acaaagaatggtggggtcaa | cacgttccacctttcctgtt | NM_001075104 | 237 |
| A_15_P1561 | *tnfsf13b* | atggcaggtgggtctaaagc | attgccgaccacgttcttct | NM_0011135 | 181 |

The genes selected from the available sequences at GenBank were associated with activation-induced cytidine deaminase (*aicda*), cytochrome b5 domain containing 1 (*cyb5d1*), ectodysplasin A receptor (*edar*), intelectin 2 (*intl2*), opioid growth factor receptor-like 2 (*ogfrl2*) and tumor necrosis factor (ligand) superfamily, member 13b (*tnfsf13b*).
